# Assessing Knowledge and Perception Regarding Antimicrobial Stewardship and Antimicrobial Resistance in University Students of Pakistan: Findings and Implications

**DOI:** 10.3390/antibiotics10070866

**Published:** 2021-07-16

**Authors:** Iltaf Hussain, Nisa Yousaf, Sana Haider, Pervisha Jalil, Muhammad Usman Saleem, Imran Imran, Abdul Majeed, Anees ur Rehman, Muhammad Uzair, Muhammad Fawad Rasool, Faleh Alqahtani, Hussain Alqhtani

**Affiliations:** 1 Department of Pharmacy Practice, Faculty of Pharmacy, Bahauddin Zakariya University, Multan 60800, Pakistan; altaf9216@gmail.com (I.H.); nisayousaf7@gmail.com (N.Y.); snhaider5@yahoo.com (S.H.); vishajalil98@gmail.com (P.J.); abdulmajeed@bzu.edu.pk (A.M.); aneesurrehman@bzu.edu.pk (A.u.R.); 2 Department of Biosciences, Faculty of Veterinary Sciences, Bahauddin Zakariya University, Multan 60800, Pakistan; usmansaleem@bzu.edu.pk; 3 Department of Pharmacology, Faculty of Pharmacy, Bahauddin Zakariya University, Multan 60800, Pakistan; imran.ch@bzu.edu.pk; 4 Department of Pharmaceutical Chemistry, Faculty of Pharmacy, Bahauddin Zakariya University, Multan 60800, Pakistan; muhammaduzair@bzu.edu.pk; 5 Department of Pharmacology and Toxicology, College of Pharmacy, King Saud University, Riyadh 11451, Saudi Arabia; 6 Department of Clinical Pharmacy, College of Pharmacy, Najran University, Najran 61441, Saudi Arabia; hmhalqhtani@nu.edu.sa

**Keywords:** antimicrobial stewardship, antimicrobial resistance, knowledge, antibiotics, perceptions

## Abstract

The irrational use of antimicrobials has enormously contributed to antimicrobial resistance (AMR) globally and especially in the developing world. To assess the knowledge and perception regarding AMR and antimicrobial stewardship (AMS), a descriptive cross-sectional study was carried out in university students enrolled in pharmacy, veterinary, and biology programs by using an online self-administered questionnaire. The Chi-square and Fisher exact tests (where applicable) were performed to assess the association of the demographics with the students’ knowledge and perception regarding AMR and AMS. A total of 496 students completed the questionnaire, among which, 85.7% of the participants were familiar with the term AMR and 79.4% of the participants correctly identified a poorly designed dosing regimen as a contributing factor towards AMR. The majority of participants (57.9%) were familiar with the term AMS and 86.5% were aware of the aim of AMS. The participants showed good knowledge regarding AMR and AMS, but to further improve student knowledge and perception of AMS and AMR, it is suggested that dedicated modules on antibiotic use and AMS should be incorporated into the curricula of these undergraduate and postgraduate programs.

## 1. Introduction

Antimicrobial resistance (AMR) is a global health issue affecting both developed and developing nations [1]. The development of AMR is a natural phenomenon, but the misuse of this miracle is accelerating the resistance process [2]. Epidemiological studies have shown a direct relation between antibiotic misuse, overuse, and AMR. The overuse and misuse of antibiotics can lead to bacterial resistance caused by the removal of the drug-sensitive receptors or the production of enzymes against the drugs [3,4]. Sir Alexander Fleming in his Noble Lecture has warned that “it is not difficult to make microbes resistant to penicillin in the research laboratories by exposing them to concentration not sufficient to kill them, and the same mechanism has sporadically occurred in the body” [5]. World Health Organization (WHO) stated that antibiotics have saved millions of lives, but they are losing their efficacy due to both their overuse and their misuse [6].

The key driver for the development of AMR is the irrational use of antibiotics [7]. Irrational use can be in the form of polypharmacy (the use of multiple antibiotics per patient), the inappropriate use of self-medication (with prescription-only drugs), implementation in non-bacterial infections, and deviation from clinical guidelines [8,9]. Moreover, inadequate dosage and the inappropriate route of administration (overuse of injections when oral formulations are more appropriate) and excessive prescription by physicians due to diagnostic uncertainty also promote the irrational use of antibiotics [10]. The development of multi-drug resistant (MDR) bacteria such as superbugs can lead to poor clinical outcomes in infected patients, longer hospital stays, an increase in treatment cost, and mortality. The United Nations has published Sustainable Development Goals (SDG) to serve as a global blueprint for a better, more equitable, and more sustainable life. The continuing AMR emergency limits the attainment of many SDGs [11]. The prevalence of the irrational use of antibiotics is high in developing countries compared to developed countries. Studies carried out in eastern and southern Europe reported the prevalence of the irrational use of antibiotics as approximately 19% and 30% respectively [5,8,9,12], while in developing countries, the range of this prevalence was 75% to 100% [13,14,15,16].

To overcome AMR, antimicrobial stewardship (AMS) may play a pivotal role by promoting the rational use of antibiotics, improving patient outcomes, reducing microbial resistance, and decreasing the spread of infections due to MDR organisms [17]. There are two major approaches to AMS, i.e., pre-and post-prescription. The pre-prescription approach restricts and requires prior authorization for the use of antibiotics by a restrictive prescriptive authority (RPA), while the post-prescriptive approach uses prospective review and feedback by reviewing the current antibiotic orders and providing the clinician with the recommendation to continue, adjust, change, or discontinue the therapy based on the available results and clinical characteristics of the patients [18]. Studies have suggested that the pre-prescription approach has a significant effect on prescription-associated antibiotic costs in a specific restricted group, but it increases the use of antibiotics that are not restricted [19,20]. The implementation of the post-prescription approach has resulted in a decrease in antibiotic use through rationalization of prescriptions and has led to improved clinician satisfaction [21,22].

Pakistan is a developing country and ranks third in the consumption of antibiotics among low- and middle-income countries [22]. About 35,000 patients use antibiotics in Pakistan per day [22,23]. However, the sale of non-prescribed antibiotics is completely restricted by national drug policy (NDP) [24]. Regardless, the self-medication using antibiotics is highly prevalent in Pakistan [24,25]. Moreover, it is important to assess the knowledge and perception of university students, especially those studying in pharmaceutical, veterinary and biological sciences, as they are the ultimate future of the healthcare system as pharmacists, veterinarians, and microbiologists/biotechnologists. Keeping this in view, the current study was conducted to assess the knowledge and perception regarding AMR and AMS among the enrolled students in at the largest university in Southern Punjab, Pakistan.

## 2. Results

### 2.1. Demographic Information of the Participants

A total of 496 out of 573 participants recorded their online responses. The response rate was 86.5%. The majority of the respondents were male (57.5%) of single marital status (96.8%) and fell within the age range of 18–23 years of age (53.5%). Most of the participants were from pharmaceutical sciences (45.8%) and had an undergraduate level of education (90.5%). The demographic details can be seen in Table 1.

### 2.2. Knowledge of Antimicrobial Resistance

Regarding knowledge on AMR, most of the participants were familiar with the term AMR (85.7%), and the majority of the participants correctly identified a poorly designed dosing regimen as a contributing factor towards AMR (79.4%). More than three-quarters of the participants considered the usage of broad-spectrum antibiotics as a promotor for AMR (87.3%). More than half of the participants correctly identified that antibiotics cannot kill viruses (59.4%). The veterinary students showed good knowledge regarding the contribution of poorly designed dosing regimens to AMR (*p* = 0.003) and as well as the contribute of the use of antibiotics for viruses/viral diseases (*p* = 0.006) compared to the students of pharmaceutical and biological sciences. The knowledge of the participants is detailed in Table 2.

### 2.3. Perception about Antibiotic Resistance

Most of the participants were aware that the irrational use of antibiotics can harm the patient (86%), and less than two-quarters of the participants believed that the use of antibiotics is inappropriate in Pakistan (46.3%). The majority of the participants had the perception that broad-spectrum antibiotics are being used unnecessarily (66.5%). The participants provided positive feedback on following the appropriate duration of antimicrobials usage (84.1%) and considered that the use of antibiotics should be reduced (76.4%). A positive perception was shown by the pharmacy students regarding the inappropriate use of antibiotics in Pakistan (*p* ≤ 0.001) and irrational use of broad-spectrum antibiotics (*p* ≤ 0.001) compared to students from veterinary and biological sciences (Table 3).

### 2.4. Knowledge regarding Antimicrobial Stewardship

The participants were found to have a good familiarity of the term AMS in the current study (57.9%). Most of the participants knew the aim of AMS (86.5%) and correctly identified the role of the AMS approach in reducing antimicrobial resistance (80.4%), as shown in Table 4. Pharmacy students reported good knowledge regarding their awareness of AMS (*p* ≤ 0.001) and its aims and objectives (*p* = 0.006) compared to students from veterinary and biological sciences (Table 4).

### 2.5. Perception about Antimicrobial Stewardship

The majority of the participants considered that knowledge regarding antimicrobial usage is important for improved patient care (74.4%) and that this role can be played by the pharmacist (86.5%). Most of the participants had a perception that the incorporation of an AMS program can ensure the therapeutic efficacy of antibiotics (55.2%). The pharmacy students had a positive perception regarding the role of the pharmacist in awareness of antibiotics use (*p* = 0.001) and the incorporation of AMS in the health system (*p* = 0.001). The details are shown in Table 5.

**Figure 1:**
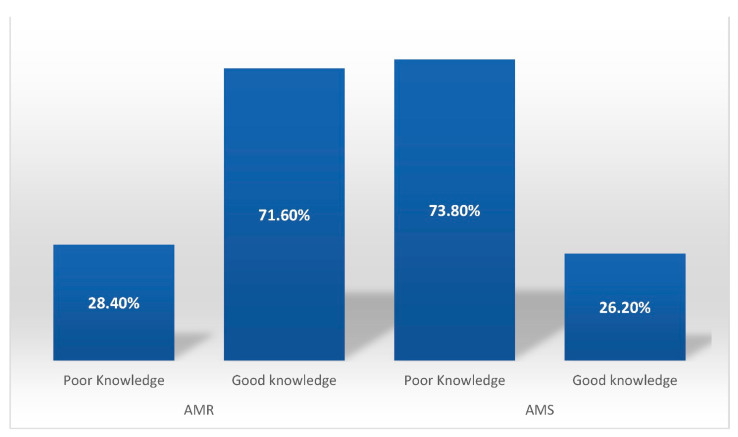
The knowledge of the study participants regarding antimicrobial resistance (AMR) and antimicrobial stewardship (AMS).

The veterinary students showed good knowledge regarding AMR and AMS compared to the pharmacy and biological sciences students (*p* ≤ 0.001 and *p* = 0.002, respectively). The antimicrobial knowledge of males was good as compared to their counterparts (*p* = 0.01), and detail can be seen in Table 6. Overall, the participants showed good knowledge regarding AMR (71.6%), but their knowledge regarding AMS was poor (73.8%). The details can be seen in Figure 1.

## 3. Discussion

The current study was conducted to assess the knowledge and perception of university students about antimicrobial resistance and stewardship. The respondents showed good knowledge, and their attitudes and perceptions were positive toward minimizing the AMR.

It has been well established that the development of AMR can be affected by antibiotic dosing regimens, as evident from the results of in vitro and animal experiments. These studies show that AMR is promoted by the prescribing of inappropriate antibiotic dosing regimens [3,26,27]. In the current study, 79.4% of the participants correctly identified poorly designed dosage regimens as a promotor for AMR. This finding was comparable with the study reported from Malaysia, where 96% of the pharmacy students who participated highlighted that the inappropriate use and poorly designed regimen of antibiotics can cause AMR [28]. The practice of poor dosage regimens is prevalent in Pakistan, as indicated in the previously reported study, which shows that only 32.3% of the participants received an appropriate dosing regimen [29]. Moreover, 87.3% of the participants considered the empirical use of broad-spectrum antibiotics as a contributing factor to AMR. This value was higher compared to a previous study conducted in Pakistan, which showed that 63.9% of pharmacy students were knowledgeable about this contributing factor [30].

Antimicrobial sensitivity and patient compliance are the key determinants for the efficacy of antimicrobial therapy [31]. Compliance is greatly affected by the fear of adverse effects, quitting treatment upon improvement of the condition, affordability issues due to high drug prices, and inadequate instructions from the pharmacist [31]. In the current study, 71.9% of the participants believed that poor adherence to a prescribed medication can lead to AMR. Moreover, 84.1% of the participants gave importance to following the appropriate duration of an antimicrobial therapy to avoid the development of the resistance. This finding was high compared to a previous report from Pakistan [32].

In the current study, 86.5% of the participants were aware of the aim of AMS. This was much higher than a previously reported cross-sectional study from three Asian countries (Indonesia, Malaysia, and Pakistan), which stated that 64.5% of the participants correctly identified the goals of AMS [33]. Most of the participants (80.4%) were knowledgeable about the nature of AMS and agreed that it is a multifaceted approach to prevent AMR. This finding was in line with previously reported data from Pakistan, where 83.5% of the participants were aware of the nature of AMS [32].

A total of 86.3% of the respondents recommended that a strong knowledge of antimicrobial usage is important for better patient care. It has been previously reported in Pakistan that 84% of participants favored the importance of knowledge of antimicrobial usage [32]. The current study findings showed that 86.5% of the participants favored the role of the pharmacist in the awareness of correct antimicrobial use. In the previous report, 84.5% of the participants favored the role of the pharmacist in AMS, which was consistent with our study [33]. Moreover, 86.5% of the participants were in favor that AMS should be incorporated into the health care system. Community pharmacists are in direct contact with the patients in their communities and therefore, could play an intrinsic role in reducing the rise of AMR by advising patients not to use antibiotics for self-limiting infections [34,35].

The Global Action Plan on AMR highlights the importance of training all healthcare professionals, including human and animal health professions, as they have important roles to play in keeping antibiotics effective [11]. The perceptions of the veterinary students regarding antimicrobial use may be different from the pharmacy and biological sciences students, as the veterinary sciences are focused on the non-human use of antibiotics [36]. It is important to mention that the good knowledge and positive perceptions of the veterinarians regarding the use of the antibiotic, AMR, and AMS is needed, as the irrational and inappropriate use of antibiotics in animals is also contributing towards the AMR [37].

### 3.1. Recommendations

From the findings of this study, it is suggested that a dedicated module on antibiotic use and AMS should be incorporated in the Pharm-D. (Doctor of Pharmacy) and DVM (Doctor of Veterinary Medicine) syllabi. Moreover, a post-graduate diploma on AMS should be offered for the graduate students who are involved in the prescribing and dispensing of antibiotics. Despite the endorsement of the national action plan by the Pakistan Ministry of National Health Services and coordination for the problem of AMR, there is a need for extensive awareness regarding antibiotic use. Pharmacists, especially community pharmacists, should take responsibility for creating awareness against the self-medication of antibiotics and their over and misuse. The community pharmacist can win this fight by advising and counseling patients and the public on the rational use of antibiotics. The WHO and UN should incorporate and prioritize the SDGs regarding antibiotics and incorporate the indicators that can help in achieving these goals as well.

### 3.2. Study Limitations

The current study may be subject to many limitations. First, the knowledge questions were based on recall, which may cause recall bias. Second, the study participants were from one geographical region of Pakistan, therefore the results cannot be generalized to the whole population. Third, assessment bias cannot be ignored, as the study questions may not necessarily cover all the topics related to AMR and AMS. Last, the study may also be associated with self-reporting bias even though the responses were voluntary.

## 4. Materials and Methods

### 4.1. Study Design and Settings

For the current study, a cross-sectional approach was used. The study setting was Bahauddin Zakariya University (BZU), Multan, Pakistan, which is the largest higher education institute in Southern Punjab [38]. Students enrolled in pharmacy, veterinary, and biology programs were selected for the current study. The study was carried out between February, and March 2021.

### 4.2. Study Instrument

A self-administered questionnaire was constructed for the survey after a review of the literature [21,23,26]. The questionnaire was divided into five sections. The first section comprised demographics including age, faculty, gender, marital status, and education level. In the second and third sections, participants were asked about their knowledge and perception of AMR. In the fourth and fifth sections, the participant’s knowledge and perception of AMS were assessed. All of the responses were collected based on “Yes”, “No” and “I don’t know” answers. The correct responses were assigned 1 point, and the incorrect response was assigned o points. The participants who gave ≥50% correct answers in both knowledge domains were considered to have good knowledge, and those with less than 50% were considered to have poor knowledge.

A pilot study was conducted by recruiting 40 participants from the selected faculties. A Cronbach alpha test was used to assess the reliability and internal consistency of the questionnaire. The value of the reliability coefficient was 0.82, which was in an acceptable range. The pilot study participants were excluded from the final analysis.

### 4.3. Data Collection

The questionnaire was designed using Google Forms (Google LLC, CA, USA). An online link was created and shared with each class representative (CRs) of the selected departments, and the CRs were responsible for sharing this link with their class fellows. It took 5–7 min to complete the survey. The statistical package for social sciences (SPSS Inc., version 25, IBM, Chicago, IL, USA) was used to perform the statistical analysis. The Checklist for Reporting Results of Internet E-Surveys (CHERRIES) guidelines were followed for online data collection [39].

### 4.4. Ethical Approval

The study was performed following the Declaration of Helsinki. The participant was briefed about the study objective, volunteer participation, and the right to withdraw. Online informed consent was taken from the participants before participation in the study. Ethical approval was obtained from the Department of Pharmacy Practice, Faculty of Pharmacy, BZU, Multan, Pakistan (ACAD/PRACT/21/05). The current study was presented according to The Strengthening the Reporting of Observational Studies in Epidemiology (STROBE) guidelines [40].

### 4.5. Statistical Analysis

The normality of the data was assessed using the Kolmogorov–Smirnov and the Shapiro–Wilk tests. The categorical variables were presented as frequencies and percentages. The Chi-square test and the Fisher exact test (where applicable) were used to assess the association of demographics variables (independent) with study questions (dependent). The Bonferroni correction was used, as the Chi-square was utilized repeatedly for multiple analyses. The *p*-value was set as statistically significant at <0.05.

## 5. Conclusions

The current study showed an overall good knowledge among the respondents, but there is still a knowledge gap in some aspects of AMR and AMS, as some of the respondents supported the use of antibiotics for viruses. Moreover, the growing trend of AMR should be treated as a “Global Emergency”, and concrete steps have to be taken by international and national health agencies to address this issue. Lastly, the Higher Education Commission of Pakistan should focus on designing AMR and AMS courses and training for undergraduate biological and medical sciences students. 

## Figures and Tables

**Table 1:** Demographic characteristics of the participants.

		N	%
Age	18–23	267	53.5
	>23	229	46.5
Department/faculty	Pharmaceutical sciences	227	45.8
	Veterinary sciences	131	26.4
	Biological sciences	138	27.8
Gender	Male	285	57.5
	Female	211	42.5
Marital status	single	480	96.8
	married	16	3.2
Education level	Undergraduate	449	90.5
	Postgraduate	47	9.5

**Table 2:** Participant’s knowledge about antimicrobial resistance.

		Overall N (%)	Pharmaceutical Sciences	Veterinary Sciences	Biological Sciences	*p*-Value
			N (%)	N (%)	N (%)	
Do you know about the term “antimicrobial resistance”?	yes	425 (85.7)	121 (92.4)	188 (82.8)	116 (84.4)	0.12
	no	46 (9.3)	5 (3.8)	26 (11.5)	15 (10.9)	
	maybe	25 (5)	5 (3.8)	13 (5.7)	7 (5.1)	
Do you know poorly designed dosing regimens can contribute to antimicrobial resistance?	yes	394 (79.4)	118 (90.1)	170 (74.9)	106 (76.8)	**0.003**
	no	55 (11.1)	9 (6.9)	26 (11.5)	20 (14.5)	
	maybe	47 (9.5)	4 (3.1)	31 (13.7)	12 (8.7)	
Do you know the usage of broad-spectrum antibiotics promotes antimicrobial resistance?	yes	433 (87.3)	121 (92.4)	194 (85.5)	118 (85.5)	0.09
	no	27 (5.4)	1 (0.8)	17 (7.5)	9 (6.5)	
	maybe	36 (7.3)	9 (6.9)	16 (7)	11 (8.0)	
Do you know that antibiotics can kill viruses?	yes	134 (27.1)	21 (16.2)	72 (31)	41 (29.7)	**0.006**
	no	294 (59.4)	95 (73.1)	123 (54.2)	76 (55.1)	
	maybe	67 (13.5)	14 (10.8)	32 (14.1)	21 (15.2)	

Chi-square of independence was applied where cell count was > 5 and Fisher exact test were applied where cell count < 5. The bold values were statistically significant at *p* ≤ 0.05.

**Table 3:** Participant’s perception of antimicrobial resistance (AMR).

		Overall N (%)	Pharmaceutical Sciences	Veterinary Sciences	Biological Sciences	*p*-Value
			N (%)	N (%)	N (%)	
Do you think irrational use of antibiotics can harm the patient?	yes	433 (87.3)	121 (92.4)	194 (85.5)	118 (85.5)	0.09
	no	27 (5.4)	1 (0.8)	17 (7.5)	9 (6.5)	
	maybe	36 (7.3)	9 (6.9)	16 (7.0)	11 (8.0)	
Do you think casual/common use of antibiotics in Pakistan is appropriate?	yes	217 (43.8)	48 (36.9)	121 (53.3)	48 (34.8)	**<0.001**
	no	229 (46.3)	78 (60)	77 (33.9)	74 (53.6)	
	maybe	49 (9.9)	4 (3.1)	29 (12.8)	16 (11.6)	
Do you think broad-spectrum anti-bacterials are used unnecessarily when narrow-spectrum antibiotics are available?	yes	330 (66.5)	109 (83.2)	137 (60.4)	84 (60.9)	**<0.001**
	no	77 (15.5)	9 (6.9)	47 (20.7)	21 (15.2)	
	maybe	89 (17.9)	13 (9.9)	43 (18.9)	33 (23.9)	
Do you think poor patient adherence to prescribed antibiotics can be a cause of AMR?	yes	356 (71.9)	110 (84.6)	151 (66.5)	95 (68.8)	**0.003**
	no	66 (13.3)	9 (6.9)	33 (14.5)	24 (17.4)	
	maybe	73 (14.7)	11 (8.5)	43 (18.9)	19 (13.8)	
It is important to follow the appropriate duration of antimicrobials to prevent the development of resistance?	yes	417 (84.1)	120 (91.6)	183 (80.6)	114 (82.6)	**<0.001**
	no	35 (7.1)	6 (4.6)	19 (8.4)	10 (7.2)	
	maybe	44 (8.9)	5 (3.8)	25 (11.0)	14 (10.1)	
Do you think antibiotic use should be reduced?	yes	379 (76.4)	117 (89.3)	153 (67.4)	109 (79.0)	**<0.001**
	no	69 (13.9)	7 (5.3)	40 (17.6)	22 (15.9)	
	maybe	48 (9.7)	7 (5.3)	34 (15.0)	7 (5.1)	

Chi-square of independence was applied where cell count was > 5 and Fisher exact test were applied where cell count was < 5. The bold values were statistically significant at *p* ≤ 0.05.

**Table 4:** Participant’s knowledge about antimicrobial stewardship (AMS).

		Overall N (%)	Pharmaceutical Sciences	Veterinary Sciences	Biological Sciences	*p*-Value
			N (%)	N (%)	N (%)	
Do you know about AMS?	yes	158 (31.9)	49 (37.4)	82 (36.1)	27 (19.6)	**<0.001**
	no	287 (57.9)	64 (48.9)	119 (52.4)	104 (75.4)	
	maybe	51 (10.3)	18 (13.7)	26 (11.5)	7 (5.1)	
AMS aims to optimize antimicrobial use.	yes	427 (86.3)	119 (91.5)	186 (81.9)	122 (88.4)	**0.006**
	no	37 (7.5)	7 (5.4)	21 (9.3)	9 (6.5)	
	maybe	31 (6.3)	4 (3.1)	20 (8.8)	7 (5.1)	
AMS is the key component of a multifaceted approach for preventing the emergence of antimicrobial resistance.	yes	429 (86.5)	126 (96.2)	192 (84.6)	111 (80.4)	**0.006**
	no	36 (7.3)	1 (0.8)	17 (7.5)	18 (13)	
	maybe	31 (6.3)	4 (3.1)	18 (7.9)	9 (6.5)	

Chi-square of independence was applied where cell count was > 5 and Fisher exact test were applied where cell count was <5. The bold values were statistically significant at *p* ≤ 0.05.

**Table 5:** Participant’s perception of antimicrobial stewardship (AMS).

		Overall N (%)	Pharmaceutical Sciences	Veterinary Sciences	Biological Sciences	*p*-Value
			N (%)	N (%)	N (%)	
Do you think strong knowledge and awareness about correct anti-microbial use is important for better patient care?	yes	369 (74.4)	106 (80.9)	171 (75.3)	92 (66.7)	0.10
	no	45 (9.1)	5 (3.8)	19 (8.4)	21 (15.2)	
	maybe	82 (16.5)	20 (15.3)	37 (16.3)	25 (18.1)	
Do you think pharmacists can play a role in awareness of correct antimicrobial usage?	yes	429 (86.5)	117 (89.3)	192 (84.6)	120 (87)	**0.001**
	no	24 (4.8)	3 (2.3)	15 (6.6)	6 (4.3)	
	maybe	43 (8.7)	11 (8.4)	20 (8.8)	12 (8.7)	
AMS should be incorporated into the healthcare system.	yes	369 (74.4)	106 (80.9)	171 (75.3)	92 (66.7)	**0.001**
	no	45 (9.1)	5 (3.8)	19 (8.4)	21 (15.2)	
	maybe	82 (16.5)	20 (15.3)	37 (16.3)	25 (18.1)	
Do you think hospital pharmacist is an essential element of AMS?	yes	399 (80.4)	117 (89.3)	173 (76.2)	109 (79)	0.47
	no	41 (8.3)	7 (5.3)	21 (9.3)	13 (9.4)	
	maybe	56 (11.3)	7 (5.3)	33 (14.5)	16 (11.6)	
Implementation of AMS can ensure therapeutic efficacy of antibiotics and reduce antimicrobial resistance?	yes	274 (55.2)	56 (42.7)	148 (65.2)	70 (50.7)	**<0.001**
	no	92 (18.5)	40 (30.5)	20 (8.8)	32 (23.2)	
	maybe	130 (26.2)	35 (26.7)	59 (26)	36 (26.1)	

Chi-square of independence was applied where cell count was >5 and Fisher exact test were applied where cell count was <5. The bold values were statistically significant at *p* ≤ 0.05.

**Table 6:** Association of knowledge regarding antimicrobial resistance (AMR) and antimicrobial stewardship (AMS) with the demographics of the study participants.

		AMR Knowledge		*p*-Value	AMS Knowledge		*p*-Value
		Poor	Good		Poor	Good	
		N (%)	N (%)		N (%)	N (%)	
Age	18–23	85 (60.3)	179 (50.9)	0.05	197 (54.1)	67 (51.9)	0.66
	>23	56 (39.7)	173 (49.9)		167 (45.9)	62 (48.1)	
Department	Pharmaceutical sciences	19 (53.2)	112 (31.5)	<0.001	87 (23.8)	44 (33.8)	**0.002**
	Veterinary sciences	75 (33.3)	152 (42.8)		162 (44.3)	65 (50)	
	Biological sciences	47 (33.3)	91 (25.6)		117 (32)	21 (16.2)	
Gender	Male	89 (63.1)	194 (54.6)	0.01	203 (55.5)	80 (61.5)	0.33
	Female	50 (35.5)	161 (45.4)		162 (44.3)	49 (37.7)	
Marial status	Single	136 (96.5)	344 (96.9)	0.79	355 (97)	125 (96.2)	0.64
	Married	5 (3.5)	11 (3.1)		11 (3.0)	5 (3.8)	
Education level	Undergraduate	135 (95.7)	314 (88.5)	0.01	330 (90.2)	119 (91.5)	0.64
	Postgraduate	6 (4.3)	41 (11.5)		36 (9.8)	11 (8.5)	

The bold values were statistically significant at *p* ≤ 0.05.

## Data Availability

The data can be requested from the corresponding author upon the presentation of a valid reason.
